# Paternal postpartum depression in an obsessive personality following the COVID-19 lockdown successfully treated with Vortioxetine

**DOI:** 10.1192/j.eurpsy.2022.1291

**Published:** 2022-09-01

**Authors:** L. Orsolini, S. Pompili, V. Salvi, U. Volpe

**Affiliations:** Unit of Clinical Psychiatry, Polytechnic University of Marche, Ancona, Italy, Department Of Neurosciences/dimsc, Ancona, Italy

**Keywords:** Covid-19, lockdown, paternal postpartum depression, obsessive personality

## Abstract

**Introduction:**

A growing amount of studies investigating the mental health impact of the current COVID-19 pandemic worldwide have been recently published, even though very few studies investigating the impact of the COVID-19 outbreak and lockdown on the mental health of fathers of newborns during the COVID-19 pandemic, particularly on paternal postpartum depression (PPD).

**Objectives:**

A case report describing a 37-years-old man with an obsessive-compulsive personality who manifests the onset of a clinically relevant PPD following his wife’s delivery during the COVID-19 pandemic and the onset of obsessive symptomatology.

**Methods:**

At baseline and during a 12-months follow-up were administered the Edinburgh Postnatal Depression Scale (EPDS), Fear of COVID-19 (FCV-19-S), Coronavirus Anxiety Scale (CAS) and Y-BOCS-II (Yale-Brown Obsessive Compulsive Scale).

**Results:**

Patient was successfully treated with vortioxetine up to 20 mg/die with a significant clinical remission of depressive and obsessive symptomatology at 6 months and a maintenance therapy with vortioxetine 10 mg daily.

**Conclusions:**

PPD should be better investigated, particularly the impact of COVID-19 pandemic on mental health of fathers of newborns during the COVID-19-related situation.

**Disclosure:**

No significant relationships.

